# Unsupervised image segmentation for microarray spots with irregular contours and inner holes

**DOI:** 10.1186/s12859-015-0842-3

**Published:** 2015-12-23

**Authors:** Bogdan Belean, Monica Borda, Jörg Ackermann, Ina Koch, Ovidiu Balacescu

**Affiliations:** 1 CETATEA Research Centre, National Institute for Research and Development of Isotopic and Molecular Technologies - INCDTIM, 67 - 103 Donat, Cluj-Napoca, Romania; 2 Department of Communication, Technical University of Cluj-Napoca, Baritiu 26-28, Cluj-Napoca, Romania; 3 Molecular Bioinformatics Group, Institute of Computer Science, Faculty of Computer Science and Mathematics, Cluster of Excellence Frankfurt “Macromolecular Complexes”, Johann Wolfgang Goethe-University, Baritiu 26-28, Frankfurt am Main, Germany; 4 Department of Functional Genomics and Experimental Pathology, The Oncology Institute “Prof. Dr. Ion Chiricuta”, Cluj-Napoca, Romania

**Keywords:** Gene expression, Microarray, PDE, Clustering

## Abstract

**Background:**

Microarray analysis represents a powerful way to test scientific hypotheses on the functionality of cells. The measurements consider the whole genome, and the large number of generated data requires sophisticated analysis. To date, no gold-standard for the analysis of microarray images has been established. Due to the lack of a standard approach there is a strong need to identify new processing algorithms.

**Methods:**

We propose a novel approach based on hyperbolic partial differential equations (PDEs) for unsupervised spot segmentation. Prior to segmentation, morphological operations were applied for the identification of co-localized groups of spots. A grid alignment was performed to determine the borderlines between rows and columns of spots. PDEs were applied to detect the inflection points within each column and row; vertical and horizontal luminance profiles were evolved respectively. The inflection points of the profiles determined borderlines that confined a spot within adapted rectangular areas. A subsequent k-means clustering determined the pixels of each individual spot and its local background.

**Results:**

We evaluated the approach for a data set of microarray images taken from the Stanford Microarray Database (SMD). The data set is based on two studies on global gene expression profiles of Arabidopsis Thaliana. We computed values for spot intensity, regression ratio, and coefficient of determination. For spots with irregular contours and inner holes, we found intensity values that were significantly different from those determined by the GenePix Pro microarray analysis software. We determined the set of differentially expressed genes from our intensities and identified more activated genes than were predicted by the GenePix software.

**Conclusions:**

Our method represents a worthwhile alternative and complement to standard approaches used in industry and academy. We highlight the importance of our spot segmentation approach, which identified supplementary important genes, to better explains the molecular mechanisms that are activated in a defense responses to virus and pathogen infection.

## Background

Microarray technology is one of the most powerful tools used to generate molecular hypotheses. It allows the interrogation of the genome functionality by assessing the expression of thousands of cellular transcripts (mRNAs), even for the entire transcriptome, in a single experiment. This technology has a broad field of applications such as in cellular functionality, investigation of pathological phenotypes, characterization of molecular subtypes, and identification of markers for diagnosis, prognosis, and treatment prediction [1]. Generally, all microarray providers developed standardized protocols specific to their technology, but there is no standardized method to process the voluminous microarray data [2, 3].

Depending on microarray technology, targets are either single-stranded DNAs or RNAs labeled with fluorescent markers, as cyanine (Cy). One or two labels (e.g. Cy3, and/or Cy5) can be utilized in the same hybridization measurement, depending on microarray study design, with one (Cy3) or two colors (Cy3 and Cy5). After synthesis, the microarray targets are hybridized on a glass microarray slide with large numbers of microscopic spots. Each spot contains a short DNA sequence of 20–60 nucleotides called probes or oligonucleotides which are specific for a gene in the genome. Specific regions of interest, e.g., SNPs, CNVs or duplicates in the genome could be included in spots. After hybridization and washing, microarray slides are scanned in specific scanners with appropriate wavelengths for fluorescent markers. A TIFF image, including the intensities for every spot, will be analyzed to compute the levels of gene expression, namely how many microarray targets are hybridized to their complementary probes [4]. An accurate determination of the gene expression level is a crucial step and involves three major tasks: (1) *grid alignment*, called addressing to determine the spatial coordinates of each spot; (2) *segmentation*, to classify pixels either as foreground, representing the DNA spots, or as background; (3) *extraction of intensity*, of each spot and its individual background. Results of the image analysis are the layout of the spot array, the spot sizes and shapes, the spot intensities (i.e., gene expression levels), and the background intensity values.

The estimation of gene expression levels has to deal with noise and artifacts introduced, e.g., during the microarray printing and the hybridization processes. The automated procedures of grid alignment and spot segmentation have to yield reliable results even for spots with various shape and size. Consequently, the automated microarray image processing is subject of on-going research, and approaches apply computationally expensive techniques for unsupervised spot segmentation. Complex Gaussian scale mixture (CGSM) model in complex wavelet domain has lead to efficient noise reduction in microarray images [5]. Support vector machine (SVM) has been applied for grid alignment [6, 7] and a fully automatic griding methods have been demonstrated [8]. To eliminate the distortions introduced by scanning and hybridization assay, spatial and distributional segmentation techniques have been evaluated in [9] and [10]. Moreover, adaptive pixel clustering for variable contours has been studied [10–14]. Spatial methods, such as the Snake Fisher model [15, 16] or 3D spot modeling [17] have been introduced and Markov random field models have combined intensity and spatial information [18, 19] for the spot segmentation. An efficient classification of pixels in background and foreground has been achieved by means of geometric measures [20] and by an algorithm based on growing con-centric hexagons [21]. Based on an automated seed selection procedure, a grow-cut procedure was successfully applied for independent segmentation of each spot [22].

Here, we present a method for unsupervised spot segmentation based on partial differential equations (PDEs). Our procedure combines spatial and distributional approaches to classify pixels as pixels of the spot (foreground) or the local background surrounding the spot. Previously, preferable features of the PDE approach for the initial step of automatic grid alignment have been demonstrated [23]. The grid alignment defines rectangular areas and each of the rectangles confines a spot. In our approach, the PDE formalism was combined with a refinement based on the autocorrelation function of the spatial intensity distribution of the fluorescent light. Ellipses adapted to the rectangle areas provided an initial classification of pixels of foreground, background, and exclusion zone. A k-means clustering refined the initial classification. We evaluated the accuracy of the method by comparing our results with reference values published in the SMD public data repository.

## Methods

**Fig. 1:**
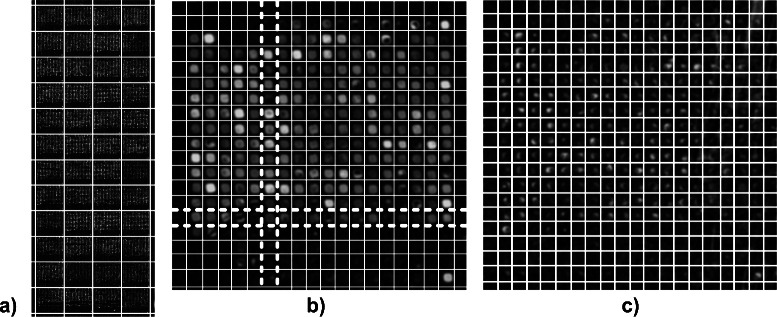
**a** The microarray image was taken from SMD database (ID 20385). The image shows the intensity of fluorescent light emitted from dye cyanine Cy3. The spots of high intensity indicate sequence-specific hybridisation of labelled DNA extracted from *Arabidopsis thaliana*. The experiment has been designed to study global transcriptional factors of hormone treatment. The detection of groups of co-localized spots is shown by the grid of the global addressing. The groups of spots are separated by the horizontal and vertical lines of the grid. **b** A sub-image of the microarray image shown in **a**). The sub-image depicts a group of 324 co-localized spots. The size of a sub-image is in round numbers 460×480 pixels. Spots of high intensity are separated by lines of a grid. Inflection points of intensity profiles were computed to align the grid, see Section Grid alignment. The horizontal and vertical lines of the griding separate rows and columns of spots. The two horizontal broken lines and the two vertical broken lines cut out a slice of the 14th row and of the 6th column of spots, respectively. **c** The procedure of grid alignment yields a stable separation even for spots with non-spherical profiles, low intensities or high background signal

Fluorescent light emitted from dye immobilized on the chip surface produces a microarray image. Conventionally, the microarray image is stored in the Tagged Image File Format (TIFF) as a two-dimensional array of intensities, *I*=(*p*_*u,v*_). The intensities, *p*_*u,v*_, are 16 bits integer with a dynamic range of 0 ≤ *p*_*u,v*_ ≤ 2^16^−1. A lower index may denote the dye, e.g. *I*_*Cy*3_, denotes a microarray image recorded of the cyanine dye Cy3. Figure 1 a shows an example of a microarray image. Let us consider a microarray image of 5550×1910 pixels size which includes a number of 15,552 bright spots, indicating the sequence-specific hybridization of labeled DNA. The image is taken form Stanford Microarray Database (SMD) and has the identification number ID 20,385. The bright spots have a diameter of, in round numbers, 15 pixels. The spots accumulate spatially to 48 groups of 324 neighbored spots each. The division of the whole image into 48 sub-images each of which containing an individual group of spots is called global addressing. Within each group the spots are located along horizontal and vertical lines (rows and columns). Our task was to identify the spots in the images and to extract the feature characteristics, such as mean intensity, background intensity, or variation of intensity, for each of the spots. The high number of pixels (in round numbers 10^7^ pixels) makes an automated image processing necessary. Our workflow included the following 4 steps: (1) preprocessing for enhancement, rotation, and global addressing, (2) grid alignment for determination of borderlines between adjacent rows or adjacent columns of spots, (3) segmentation for the classification of pixels to foreground and background, and (4) extraction of intensity features.

### Preprocessing

This step included: a logarithmic transformation, *p*_*u,v*_→*r*_*u,v*_=log_2_(*p*_*u,v*_+1), which mapped the 16 bit integer intensities, *p*_*u,v*_, to the real-valued grey scale, 0 ≤ *r*_*u,v*_ ≤ 16. Further, a global shift and uniform scaling of intensities *r*_*u,v*_ yielded an image $I^{L}=(p^{\prime }_{\text {\textit {u,v}}})$ with properties, $\min \left (p^{\prime }_{\text {\textit {u,v}}}\right)=0$, $\max \left (p^{\prime }_{\text {\textit {u,v}}}\right)=2^{16}-1$, and enhanced contrast. To adjust spots to horizontal and vertical lines, we rotated the entire image using Radon transform [6, 8]. We split the image into sub-images, each of which containing one of the spatially organized group of spots. For the splitting, we followed the strategy proposed by Angulo and Serra [24], and applied a morphological dilation operation to fuse neighbored spots. Such preprocessed sub-images $I^{\prime }=\left (p^{\prime }_{\text {\textit {u,v}}}\right)$ which contain a group of spots were the starting point for the succeeding steps, see Fig. 1 b for an example of a sub-image.

### Grid alignment

We applied a convolution with a Gaussian kernel of size *k*=3 pixel and standard deviation of *σ*=5 pixel to the image *I* ^′^. An average process along the *x* and *y* direction yielded the profiles

(1) $$ H(x)=\frac{1}{\text{dim}_{y}} \sum_{y} p^{\prime}_{x,y}\,\ \text{and}  $$

(2) $$ V(x)=\frac{1}{\text{dim}_{x}} \sum_{x} p^{\prime}_{x,y}.   $$

dim_*x*_ and dim_*y*_ were the dimensions of the sub-image (given in number of pixels). A shock filter processed the profiles H and V based on the partial differential scheme [25]

(3) $$ P^{t+1}=P^{t}-\text{sign}\left(\Delta P^{t}\right) \left|\nabla P^{t}\right|   $$

with iterations *t*=0,1,…,*t*_*f*_ and initial conditions of either *P* ^0^=*H* or *P* ^0^=*V*. The number of iterations was *t*_*f*_=50. The spatial discrete formulation of the iteration applies $\Delta P(i) \doteq P(i+1)-2P(i)+P(i-1)$ and $\nabla P(i) \doteq \min {\left (\Delta _{l}P(i),\Delta _{r}P(i) \right) }$ if both, the left derivative, ∇_*l*_ *P*(*i*)≡*P*(*i*+1)−*P*(*i*), and the right derivative, ∇_*r*_ *P*(*i*)≡*P*(*i*)−*P*(*i*−1), have equal signs. For opposite signs, the shock filter executes the identity operation, *P* ^*t*+1^(*i*)≡*P* ^*t*^(*i*). The shock filter has been designed to create ruptures at inflection points of the profile. A detailed discussion of the shock filter approach can be found in [23]. During shock filter iteration, the profiles converged to piece-wise constant functions. The iteration produced discontinuities, i.e., steps, at positions *h*_1_,*h*_2_,…,*h*_*m*_ and $v_{1},v_{2}, \ldots, v_{m^{\prime }}$ of the inflection points of the horizontal intensity profile, H, and the vertical intensity profile, V, respectively. The ordered sequence *h*_1_,*h*_2_,…,*h*_*m*_ contained left and right positions, *h*_2*i*_,*h*_2*i*+1_, for each gap between two adjacent columns of spots. The center, *x*_*i*_=(*h*_2*i*_+*h*_2*i*+1_)/2, is located centrally between adjacent maxima of the profile H. Similarly, we computed positions, *y*_*i*_=(*v*_2*i*_+*v*_2*i*+1_)/2, to separate rows of spots. Horizontal and vertical lines at the computed positions, $y_{1},y_{2}, \ldots, y_{m^{\prime }/2}$ and *x*_1_,*x*_2_,…,*x*_*m*/2_, respectively, define a grid on the sub-image image *I* ^′^. The grid separates a spot from its neighbors and cuts the image into small rectangles, each of which contains a single spot; for an illustration, see Fig. 1 b.

### Spot segmentation using autocorrelation driven PDE and k-means clustering

**Fig. 2:**
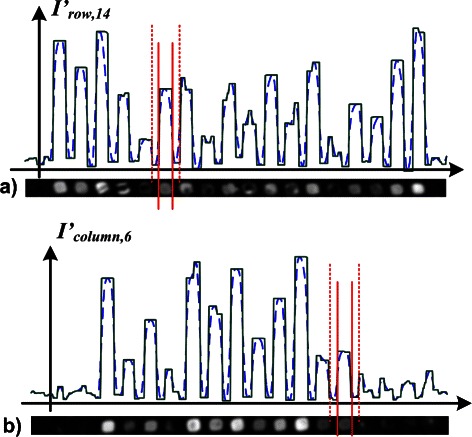
**a** The image of the 14th row of spots from Fig. 1 b is depicted at the bottom. The intensity profile before (*broken line*) and after (*solid line*) shock filter iteration is represented on top of the image. Vertical lines are drawn in red at positions of inflection points. The solid lines indicate border lines for the spot in row 14 and column 6. The broken lines confine its local background area. **b** The image of the 6th column of spots from Fig. 1 b is shown at the bottom (rotated counter clockwise by 90°). The intensity profile before (*broken line*) and after (*solid line*) shock filter iteration is depicted on top of the image. Vertical lines are drawn in red at positions of inflection points. The solid lines indicate border lines for the spot in row 14 and column 6. The broken lines confine its local background area

The segmentation consisted of three steps: (1) cutting the image, (2) initial classification into foreground, background, and exclusion zone based on an approximation of spots by ellipses, and (3) refinement of the classification by local clustering. In the first step, we cut the image *I* ^′^ into sub-images, $I^{\prime }_{\text {\textit {row,i}}}$ and $I^{\prime }_{column,j}$. The sub-image $I^{\prime }_{\text {\textit {row,i}}}=\left (p^{\prime }_{\text {\textit {u,v}}}\right)$ with *y*_*i*_≤*v*≤*y*_*i*+1_ was the horizontal slice of *I* ^′^ that contained the i.th row of spots, see Fig. 2 a. The sub-image $I^{\prime }_{\text {\textit {row,i}}}$ contained the spots in the i.th row. In the same way, the sub-image, $I^{\prime }_{\text {\textit {row,j}}}=\left (p^{\prime }_{\text {\textit {u,v}}}\right)$ with *x*_*j*_≤*u*≤*x*_*j*+1_, contained the spots in the j.th column, see Fig. 2 b. In the second step, we computed a profile P for each slice of the image, applied the shock filter iteration, and determined the positions of the inflections points. Here, we followed the approach outlined in the Methods section, description of the grid alignment approach. We assigned the positions of the inflection points to borders of an individual spot, see Figs. 2 a and b. The tight borders, *h*_*l*_<*h*_*r*_, fulfilled the 3 conditions:

(4) $$ \begin{aligned} | \nabla P(h_{l}) | > thr \ \ & \text{and }\ | \nabla P(h_{r}) | \ \quad > thr \\ x_{l} < h_{l} < x_{r} \ \ & \text{and }\ \nabla P(h_{l}) \!\qquad> 0 \\ x_{l} < h_{r} < x_{r} \ \ & \text{and }\ \nabla P(h_{r}) \!\qquad< 0. \end{aligned}  $$

**Fig. 3:**
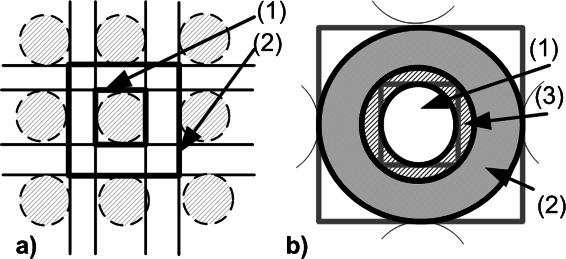
**a** The schematic diagram shows rectangles, *R*_*small*_ (1) and *R*_*big*_ (2). *R*_*small*_ embeds only the spot in the middle of the field, whereas *R*_*big*_ encloses additionally the local background area. **b** A blow up of the two rectangles, *R*_*small*_ and *R*_*big*_, lead to the definition of the three inscribed ellipses *E*_*F*_, *E*_*B*_, and *E*_*E*_. The defined ellipses show the areas of foreground (1), background (2), and exclusion zone (3) pixels

*x*_*l*_ and *x*_*r*_ were the positions of left and right horizontal grid lines, respectively, and the threshold *thr* was 30 *%* of the average intensity of the profile, P. For some spots of very low intensity, the method failed to determine inflection points, and the sequence of computed inflection points had gaps. To fill these gaps, we took advantage of the periodicity of the intensity profile and computed the autocorrelation curve. The first maximum of the autocorrelation curve determined the typical distance between spots, and hence, we filled the gap by periodic continuation of the position of inflection points. The inflection points determine the size and coordinates of rectangles, *R*_*small*_ and *R*_*big*_, see Fig. 3 a. Whereas *R*_*small*_ embeds only the spot, the bigger rectangle *R*_*big*_ includes additionally the local background area between the spot and its neighboring spots.

For an initial classification of pixels, we used three ellipses, *E*_*F*_, *E*_*B*_, and *E*_*E*_, adapted to the rectangles, *R*_*big*_, and *R*_*small*_, respectively. For an illustration we refer to Fig. 3 b. *E*_*F*_ is the ellipse with the maximum area located inside the small rectangle *R*_*small*_. *E*_*E*_ has the same centre, but its major and minor radii are 3 pixels larger. *E*_*B*_ is the ellipse with maximum area located inside the big rectangle *R*_*big*_. Pixels inside ellipse, *E*_*F*_, are assigned to the foreground, and pixels inside ellipse, *E*_*B*_, but outside ellipse, *E*_*E*_, are assigned to background. Pixels inside ellipse, *E*_*E*_, but outside ellipse, *E*_*F*_, represent an exclusion zone between foreground and background, see Fig. 3 b.

The initial classification assumed a well-shaped ellipsoid spot and did not account for irregular contours or inner holes. In a third step, we refined the initial classification and applied k-means clustering. The k-means clustering assigned the intensity of a pixel to one of the two groups: foreground (high value), or background (low value). It was applied locally for each spot, considering pixels inside ellipse *E*_*E*_, from both *I*_*Cy*3_ and *I*_*Cy*5_ images. Consequently, the clustering procedure yields two sets of pixels for the same microarray spot, denoted by *S*_*Cy*3_ and *S*_*Cy*5_, corresponding to the *I*_*Cy*3_ and *I*_*Cy*5_ image, respectively. Each set is defined as the pairs of pixel indices *S*={(*i,j*)} relative to the microarray image I, with pixel intensity value *p*(*i,j*) assigned by the clustering procedure to the foreground pixels group (high pixel intensity values). The union of the two sets *S*_*Cy*3_∪*S*_*Cy*5_ contains pixels that are called foreground of the spot (*i,j*) in both *I*_*Cy*3_ and *I*_*Cy*5_ images.

### Extraction of intensity features

For each spot, we computed the median intensity, *F*_*u*_, of the foreground and the median of intensity, *B*, of its local background. The background corrected intensity is given by the difference, *F*=*F*_*u*_−*B*. For a comparison study, the background corrected intensity, *R*, of a spot in the image, *I*_*Cy*3_, has to be compared with the background corrected intensity, *G*, of the spot at identical location in the reference image, *I*_*Cy*5_. We computed the *R* and *G* values for each of the spots in our test data set. To correct for intensity-dependent patterns in the (*R,G*) data, we applied the standard scatter plot smoother “lowess” of Cleveland and Devlin [26], with linear fit and window size of 20 %.

Conventionally, the ratio *r*=*R*/*G* measures the change of gene expression compared to a reference. Alternatively, the change of gene expression can be measured for a spot by a regression ratio *rR* [27]. The regression ratio *rR* is the slope of the linear fit through a scatter plot. The scatter plot has a point (*r,g*) for each pixel inside the surrounding ellipse, *E*_*B*_ (i.e., foreground, exclusion zone, and background). The values, *r* and *g*, are the raw intensities of the red and green channel, i.e., the intensities in the images, *I*_*Cy*3_, and, *I*_*Cy*5_, respectively. Most preferably, the value of regression ratio, *rR*, is identical to the value of the ratio, *r*. Since the regularity of the spot and spatial homogeneity of the intensity distribution inside the spot influence the fit, we computed the coefficient of determination, *R* ^2^, of the linear fit function with the points in the scatter plot to indicate the quality of a spot [28]. A value *R* ^2^=1 is the best value whereas *R* ^2^=0 is the worst result.

## Results and discussion

### Data set of images

We selected two reference data sets, each set composed of 8 images from the SMD data repository (http://smd.princeton.edu/) For the first data set, the SMD experiment IDs are 20385, 20391, 20392, and 20395 whereas for the second data set the SMD experiment IDs are 26409, 26415, 26425, and 26426. Moreover, in case of the first set, each image has the size of 5550×1910 pixels and contains 48 spot groups with 324 spots per group. The second data set contains images of 4000×1944 pixels size with 32 spot groups and 372 spots per group. Each of the two sets is organized in four pairs, (*I*_*Cy*3_,*I*_*Cy*5_), of images. Intensity features of the spots in the images have been determined, using the Molecular Devices GenePix software (https://www.moleculardevices.com), and have been made available in the SMD data repository for the entire dataset. In case of the first data set, the image pairs are the results of four experiments in a study of the global transcriptional factors for a hormone treatment of *Arabidopsis thaliana* (http://www.arabidopsis.org, Microarray Experiment Category: Hormone treatement, Experiment name: Transcriptional profiling of WT, axr3-1 and arx3-1R4). In each pair of images, the image of cyanine dye, Cy3, is the reference image, whereas image of cyanine dye, Cy5, intents to capture the incremental change induced by the treatment of *Arabidopsis thaliana* with the auxin indole acetic acid (IAA). The same data set has been analyzed previously by [23]. Considering the second data set, the image pairs are the results of four experiments describing the changes in the global gene expression profiles of susceptible Arabidopsis leaves for supporting the biotrophic fungi [29]. The image of cyanine dye, Cy3, is the reference image, whereas the image of cyanine dye, Cy5, intents to capture the changes induced by the biotrophic fungi. Regarding the quality categories of the images, the main characteristic of the entire data set is the presence of spots with irregular contour and inner whole, for which the segmentation method is addressed. Weakly expressed spots, missing rows of spots and artifacts are also present as quality categories in case of the selected images.

### Experimental results

We applied our processing pipeline to the reference data sets. Figure 1 a illustrates the detection of groups of co-localized spots in an image (ID 20391, Cy3). The groups of spots are separated by the horizontal and vertical lines of the grid. The blow ups in Fig. 1 b and c show the separation of spots within two groups of spots. Inflection points of intensity profiles were computed to align the horizontal and vertical lines of the grid, see grid alignment approach from section Methods. The procedure of grid alignment yielded a stable separation of adjacent spots even for images with spots of non-spherical profiles, spots of low intensity, and spots of high background signal.

**Fig. 4:**
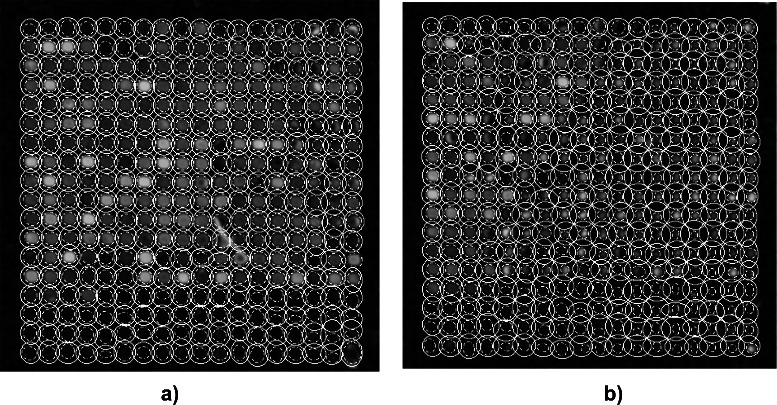
The geometrical features of each spot are approximated by a foreground ellipse *E*_*F*_ (*broken line*) and a background ellipse *E*_*B*_ (*solid line*). The approximation is shown for two groups of spots, i.e., two sub-images of image AT20391 (dye Cy3) depicted in figure panels **a** and **b** respectively. Spots of low intensity and non-spherical profiles, as well as artifacts of high background signal, make the identification and description of foreground and background area non-trivial. The ellipses show a rather high diversity in size and form but give a reasonable initial approximation of the foreground and background area of each spot

The gridding shown in Fig. 1 b and c was prerequisite to cut an image into slices of rows and columns, to compute the inflection points of intensity profiles, and to approximate each individual spot by three adapted ellipses, see the section Methods, description of the spot segmentation approach. Figures 4 a and b exemplify foreground ellipses *E*_*F*_ (broken line), and background ellipses *E*_*B*_ (solid line) for spots in two sub-images. The images in Fig. 4 a and b depict a number of spots with inner holes, spots with irregular contours, weakly expressed spots, as well as staining artifacts. For each spot in the regular pattern, the adapted ellipses of various form and size give a reasonable initial identification of the foreground and background area.

**Fig. 5:**
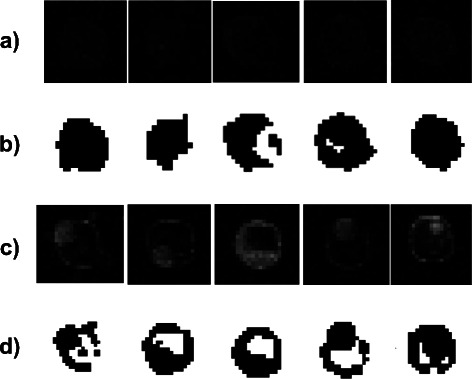
**a** The blow ups exemplify spots with low intensity. **b** The black areas are the computed foregrounds of the spots above. **c** The blow ups exemplify spots with irregular contours and inner holes. **d** The black areas are the computed foregrounds of the spots above

The approximation by ellipses illustrated in Fig. 4 a and b ignores irregular contours and inner holes of low intensity which are present in the vast majority of microarray images. The blow ups in Fig. 5 a and c exemplify spots for which a spatial characterization by ellipses is problematic, e.g., spots with low intensity, irregular contours, and inner holes. The classification of pixels inside ellipse *E*_*E*_ (foreground and exclusion zone) was refined individually for each spot by local k-clustering, see section Methods. Below the spots in Fig. 5 a and c, their corresponding foreground areas are shown in black, see Fig. 5 b and d. The computed foreground areas resemble the spatial intensity distributions of the images above. Irregular contours and holes of low intensity inside the spots have been identified correctly. Local clustering yielded a stable and preferable identification of the foreground area even in problematic cases of spatial non-homogeneous and non-spherical spots.

We determined the *R*/*G* ratios *r*, the regression ratio *rR*, and the coefficients of determination *R* ^2^ for each of the spots in our test set of microarray images. Our results were similar to the results of GenePix. The group of spots with *n* highest *R*/*G* values between our approach and GenePix data share a fraction of 60−80 *%* spots for *n*>10. For a minor fraction of, in round numbers, 6 *%* of the spots, the rank order was significantly different between two approaches (,i.e., deviation more than 30 *%* in the rank order). The values of 0.935 and 0.919 for the Pearson coefficients indicate a nearly perfect correlation between the median intensities R and G of our method and the reference values of GenePix, see Table 1. Moreover, close correlation between the coefficients of determination (Pearson coefficient 0.94) shows that the quality of spots determined using the proposed segmentation approach is very similar with the one determined using GenePix.

**Table 1:** The table exemplifies background corrected intensities, *R*, and *G*, in units of 1000 counts, the *R*/*G* ratio, the regression ration *rR*, and the coefficients of determination, *R* ^2^, for two spots, i.e., for spots no. 100 and 3260 on microarray ID 20385 shown in Fig. 1

Spot		*R*/1000	*G*/1000	*R*/*G*	*rR*	*R* ^2^
Perfect	GenePix	46.56	37.84	1.23	0.71	0.87
	Our results	51.59	40.45	1.27	0.70	0.87
		(45.73)	(37.74)	(1.21)		
Irregular	GenePix	28.63	17.68	1.61	1.41	0.76
	Our Results	65.53	37.532	1.74	1.18	0.76
		(19.29)	(11.59)	(1.66)		
Pearson correlation		0.935	0.919			0.94

Values of GenePix are compared with our results. The last line gives the Pearson correlation between GenPix and our results for all spots of an experiment (ID 20385). In parentheses are the *R* and *G* values inside the ellipses

**Fig. 6:**
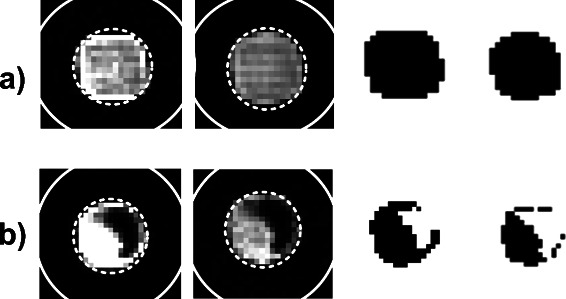
The red channel (first column) and green channel (second column) of two spots exemplify two categories of spots: **a** A perfect spot with preferable spherical and homogeneous intensity distribution. **b** An irregular spot with high contrast between foreground and background intensity. The spots with spot no. a) 100, and b) 3260 are from microarray ID 20385

For the majority of spots in the tested set of microarrays, we yielded, within insignificant fluctuations, very similar results like that in the standard approach (GenePix). The extraction of intensity features by standard methods is unproblematic for spots of spherical contour with high contrast and our data are in accordance with the reliable reference values for these spots. Figure 6 displays the red and green channel of two exemplary spots. The first spot in Fig. 6 a has a preferable spherical and homogeneous intensity distribution (perfect spot). The second spot in Fig. 6 b has an irregular contour, but nonetheless a preferable high contrast between foreground and background intensity (irregular spot). Table 1 compares our results for these spot with the reference values of GenePix.

For the perfect spot in Table 1, the median intensity, *R*, inside the ellipse, *E*_*F*_, gave an initial intensity value of, in round numbers, 45 k Counts. The ellipse, *E*_*F*_, surrounds a foreground area of 210 pixels, see Fig. 6 a. A k-means clustering was applied independently to the red and to the green channel to refine the rough, ellipse based separation of bright foreground pixels from darker background. The union of 183 foreground pixels of the red channel with the 174 foreground pixels of the green channel yielded a foreground area of 185 pixels; see Fig. 6 a for an illustration. A fraction of 6 % foreground pixels (i.e., 12 pixels) were classified as bright pixels only in the red channel. The refinement of the foreground area led to a correction of the median intensity *R* to a slightly higher value of, in round numbers, 51 k Counts. The standard intensity value of GenPix of 46 k Counts is approximately 10 % smaller than our final *R* value. The low intensity value of GenePix indicates a non-perfect gridding and an approximation of the foreground area by an ellipse that contains beside the bright foreground pixels also background pixel of low intensity. For the green channel, *G*, the intensity value of, in round numbers, 37 k Counts is within 1 % indistinguishable from the GenPix value. For the perfect spot, the differences in the intensities led to an insignificant deviation of the *R*/*G* ratio of GenePix from our *R*/*G* ratio. The deviation of both *R* ^2^ and *rR* from the GenePix values are also minor.

For the irregular spot in Table 1, the characteristics of our approach become more pronounced. For this spot the ellipse *E*_*F*_ contained 208 pixels. The median intensities of these pixels, i.e., *R*≈19 k Counts and *G*≈11 k Counts, respectively, are relatively low because of the contribution of a significantly large fraction of rather dark pixels, see Fig. 6 b. The clustering reduced the foreground area by 74 dark pixels and, hence, the median intensities of the red and green channels triple to *R*≈65 k Counts (factor 3.4) and to *G*≈27 k Counts (factor 2.4). The reference intensities of GenePix are between the median intensity inside the ellipse, *E*_*F*_, and the results of the clustering. We obtained a *R*/*G* ratio of *r*=1.74 slightly different from the reference value *r*=1.61 of GenePix. Note that our results for the median intensities inside the ellipse (values in parentheses in Table 1) yield a value of *r*=1.66 which is closer to the reference value of GenePix. Since intensity inside the ellipse is biased by a significant fraction of dark background pixels, the higher intensity ratio of the clustering (*r*=1.74) is more trustworthy.

Considering the entire data set, the median intensity values *R* and *G* determined by the proposed segmentation approach and the ones drawn from the SMD database (i.e. median intensity values computed using GenePix) were normalized using the standard lowess smoother, presented in the section Methods. The normalisation procedure is used to compensate for the effects of non-homogeneous staining of the microarray. Further on, we identified the set of *up-regulated* spots (*r*>2) using both our proposed segmentation approach and GenePix. The up-regulated spots correspond to the activated genes in the two studies on global gene expression profiles of Arabidopsis Thaliana, considered in our data sets. A discussion on the supplementary set of genes determined using our segmentation approach and their significance is presented next.

**Fig. 7:**
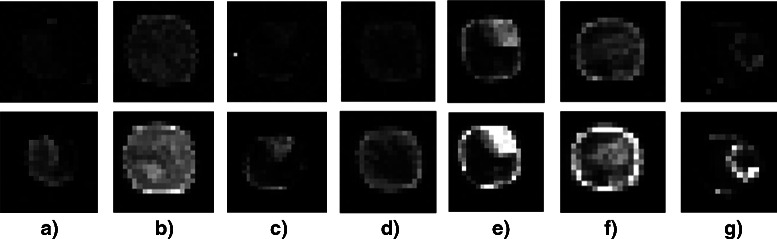
Selection of up-regulated spots that have not been identified by the standard approach of GenePix: spot no. **a** 2321, **b** 13,150, **c** 6294, **d** 4648 on microarray ID 20385, spot no. **e** 1317 and **f** 6728 on microarray ID 20391 and spot no. **g** 4422 on microarray ID 20392. Our approach allows for the irregular contours and the non-homogeneous intensity distributions of these spots. Standard approximations of the bright foreground areas based on the simple geometric forms of ellipses are problematic

The numbers of spots that are classified as up-regulated are given in Table 2. The quantities, |*A*|, |*B*|, |*A*∩*B*|, |*A*/*B*|, and |*B*/*A*|, denote the numbers of spots that are found to be up-regulated by GenePix, by our approach, simultaneously by both approaches, exclusively by GenePix, and exclusively by our approach, respectively. Our results, analyzing the *R*/*G* ratios, identified a higher number of up-regulated spots than using GenePix. Our set of up-regulated spots is, however, not a superset of the up-regulated spots of GenePix. For individual microarrays in our test set, fractions of 15 % up to 75 % of the spots that had been found up-regulated by GenPix were not confirmed by our method. Moreover, fractions of 24 – 81 % of our set of up-regulated spots have not been identified by GenePix. A question which arises is how the two segmentation methods, GenePix and the proposed one, reflect on the obtain results. GenePix software supports irregular spot detection, and you can choose to find the holes as well [30]. The proposed k-means clustering refinement within the segmentation procedure considers the local background of each spot (i.e. ellipse *E*_*E*_) and uses a distributional approach for segmentation which accounts for non-homogeneous intensity distribution, not only for holes. The advantage of our proposed method is highlighted by a selection of up-regulated spots that had been identified exclusively by our approach (see Fig. 7). The spots are characterized by irregular contours and non-homogeneous intensity distributions.

**Table 2:** The number of the up-regulated spots, *#*(*r*>2), is listed for four microarrays

ID		*#*(*r*>2)	|*A*∩*B*|	|*A*/*B*|	|*B*/*A*|
20385	GenePix |*A*|	274	181	93	60
	Our results |*B*|	241			
20391	GenePix |*A*|	33	8	25	35
	Our results |*B*|	43			
20392	GenePix |*A*|	328	257	71	54
	Our results |*B*|	311			
20395	GenePix |*A*|	9	7	2	31
	Our results |*B*|	38			
26409	GenePix |*A*|	379	298	81	98
	Our results |*B*|	396			
26415	GenePix |*A*|	171	114	27	34
	Our results |*B*|	148			
26425	GenePix |*A*|	94	47	47	80
	Our results |*B*|	127			
26426	GenePix |*A*|	229	116	113	142
	Our results |*B*|	258			

The number of up-regulated spots of GenePix is compared with the number of up-regulated spots of our approach. The number of spots that are classified as up-regulated by both approaches are given by |*A*∩*B*|. The number of spots that are classified by only one method are given by |*A*/*B*| and |*B*/*A*|, respectively

Because a microarray experiment represents an exploratory tool to investigate genes and molecular pathways, it is very important to identify very precisely all sets of genes modulated in cells of interests. Depending on their position in a molecular mechanism, each gene can be directly or indirectly activated in a cascade belonging to a particular mechanism. For example, transcription factors such as NF-kB, Jun, Fos, are involved in the activation of many genes specific for different pathways. Any lack of data in a microarray experiment could negatively influence the understanding of cellular alterations. Thereby, by lacking of the identification of some important genes, named nodal genes, the scientists could not characterize entirely the alterations that occur in the cell. In case of the first data set, using our approach, we identified more activated genes as were obtained using the GenePix software, considering the microarray experiment with ID 20385. These genes have different roles: the At1g69295 gene (index 13150) is known as mediator of biological processes [31], the At2g35980 gene (index 6294) is involved in plant response to cellular stress [32] whereas the At4g26910 gene (index 4648) is involved in metabolic processes [33].

Considering the second dataset, we also obtained a supplementary set of genes as compared with GenePix. We verified the role of these genes, in the context of microarray study design, related to susceptible Arabidopsis leaves for supporting the biotrophic fungi. We identified certain genes of interest, including At1g74520 nodal gene with index number 2110. To evaluate the importance of these genes, we further evaluate their key position based on pathway analysis assessment. It was reported that At1g74520 has a key role as a mediator of defense responses to virus and pathogen infection, through At3g50370 and At1g10390 genes [34]. As all in all, we highlight the importance of our analysis, which identified supplementary important genes, to better explains the molecular mechanisms that are activated in a defense responses to virus and pathogen infection.

We performed the calculations on a computer workstation with an Intel i5, 3.3 MHz processor and 4 GB RAM. The processing of a single microarray image took several minutes, e.g., in round numbers, 21 mins for microarray image ID 20385.

## Conclusion

We presented a novel approach for the extraction of intensity features of spots. Standard steps of image preprocessing were combined with a shock filter iteration to compute the precise position of inflection points in the vertical and horizontal intensity profiles of each individual spot. Based on the positions of inflection points, we approximated for each individual spot the foreground area, background area, and an exclusion zone between them by the intersection of adapted ellipses. We performed segmentation of the image by simple geometric objects of ellipses and this strategy turned out to be stable and reliable only for spots with spherical and homogeneous intensity distribution. For spots with irregular contour and non-homogeneous intensity distribution, this initial classification of pixels into foreground and background pixels yielded only a rough approximation that was insufficient to extract reliable values for intensity features. To overcome this drawback, we introduced a refinement step to adapt the segmentation to irregular contours as well as to dark background pixels inside a spot of bright foreground pixels. For the re-classification of the pixels inside the foreground ellipse and the background ellipse, we applied the k-means clustering method. For spots with spatial non-homogeneous intensity distribution the clustering yielded a significant rearrangement of pixels to foreground and background that, by visual inspection, fit much better to the true shape of the spots.

We tested our pipeline for a set of microarray images.

For the majority of spots, we yielded, within insignificant fluctuations, very similar results as the standard approach (GenePix). The Pearson coefficients exceeded values of 0.94 and hence, indicated a high correlation of our data (intensities) with the reference values. We extracted the set of up-regulated spots, i.e., spots with *R*/*G* ratios larger than 2, for each microarray. When comparing our results with the reference values, our approach confirmed for some microarrays up to 75 % of the reported cases of up-regulated spots in the reference data. For other microarrays the accordance dropped to 24 %, i.e., for microarray ID 20391 only 8 spots out of 33 were confirmed by our approach. Moreover, our approach identified a rather high number of up-regulated spots (22 – 81 %) that has not been reported in the reference data. Our approach computed a very precise gridding for the spots and accounted for irregular contours and inner holes in the spatial intensity distribution of the spots. As a result the obtained classification of the foreground area fits much better to the true shape of a spot; the extracted intensity features can be considered most suitable to reflect the staining of the spot. The shape and the size of the foreground area are valuable information to assess the quality of the spot and reliability of numerical results. Our method represents a worthwhile alternative and complement to standard approaches used in industry and academy. We highlight the importance of our spot segmentation approach, which identified supplementary important genes, to better explains the molecular mechanisms that are activated in a defense responses to virus and pathogen infection. The approach has to be validated in future studies, to establish its power to predict the biological significance compared to conventional methods.
